# Robotic Heller’s cardiomyotomy for achalasia: early outcomes for a high-volume UK centre

**DOI:** 10.1308/rcsann.2023.0065

**Published:** 2023-10-16

**Authors:** EJ Nevins, K Greene, S Bawa, L Horgan

**Affiliations:** Northumbria Healthcare NHS Foundation Trust, UK

**Keywords:** Achalasia, Heller’s cardiomyotomy, Robotic surgery, Outcomes

## Abstract

**Introduction:**

Heller’s cardiomyotomy (HCM) is the gold standard treatment for achalasia. Laparoscopic HCM has been shown to be effective with low rates of symptom recurrence, though oesophageal mucosal perforation rates remain high. The aim of this prospective case series is to assess the short-term complication rates and perioperative outcomes for the first cohort of patients undergoing robotic-assisted HCM for achalasia in a single high-volume UK centre.

**Methods:**

Data were collected from a prospective cohort of patients who underwent robotic HCM at a single high-volume UK centre. Outcomes were assessed using the Eckhard score, which was calculated after their routine postoperative clinic appointments.

**Results:**

Thirteen patients underwent robotic HCM during the study period; this is the second largest reported case series in the European literature. There were no intraoperative oesophageal perforations. Six patients were discharged as day cases, six patients were discharged on the first postoperative day and one patient’s hospital stay was two nights. There was a single perioperative complication of urinary retention. All patients reported improvement of symptoms following their operation, and all had a postoperative Eckhard score of less than 3, indicating their achalasia was in remission.

**Conclusions:**

This cohort has demonstrated that robotic HCM has an exceptional safety profile and results in high levels of symptom resolution, even early in the learning curve. The robotic approach may be superior to laparoscopy as it allows more precise identification and dissection of the oesophageal muscle fibres, which likely reduces the risk of inadvertent mucosal damage or incomplete myotomy.

## Introduction

Achalasia of the oesophagus is a neurodegenerative disorder characterised by impaired oesophageal peristalsis and failure of lower oesophageal sphincter relaxation. This results in obstructive symptoms that include dysphagia, regurgitation, aspiration and chronic cough.^1,2^ Although the prevalence of achalasia is only 0.4–1.2 per 100,000 population, it is the most common primary oesophageal motility disorder.^1,3,4^

The aetiology of achalasia is unknown, but histopathological findings include loss of myenteric ganglia, collagen deposition and infiltration of lymphocytes. Management of achalasia is therefore confined to palliation of symptoms by disruption of the lower oesophageal sphincter tone by medical, endoscopic or surgical methods. Medical therapies that aim to relax smooth muscle, such as calcium channel blockers or nitrates, often fail. Endoscopic pneumatic balloon dilation (EPD) of the lower oesophageal sphincter produces satisfactory results; approximately 60–85% of patients have long-term symptom relief after the first dilation, but there is a 4% perforation rate.^1,5–7^ Alternative endoscopic therapies, such as Botox, are generally considered inferior to EPD, with symptoms reoccurring six months after treatment.^8,9^ Peroral endoscopic myotomy can also be considered for the treatment of achalasia, but may result in increased incidence of reflux oesophagitis when compared with other operative treatment strategies.^10^

Heller’s cardiomyotomy is the gold standard treatment for achalasia. It is reported to have superior outcomes when compared with endoscopic management strategies.^11–13^ Laparoscopic Heller’s cardiomyotomy (LHCM) has been shown to be effective with recurrence of symptoms in 10–25% of patients, but has a reported oesophageal mucosal perforation rate of 4–20%.^14–18^

Recent meta-analyses of studies from North America, where robotic surgery has been more readily adopted, have demonstrated non-inferiority of robotic Heller’s cardiomyotomy (RHCM) compared with LHCM with regards to the majority of postoperative complications and outcomes.^19,20^ It is suggested that RHCM may reduce intraoperative oesophageal perforation rates; this has been attributed to improved optics, instrument stability and improved instrument articulation, achieved with the robotic platforms.^21–23^ These advantages may also aid in achieving complete muscle fibre disruption during the myotomy, which is necessary to ensure resolution of symptoms.^18^

The aim of this prospective case series is to assess the short-term complication rates, and perioperative outcomes, for the first cohort of patients undergoing RHCM for achalasia in a single high-volume UK centre.

## Methods

This is a prospective cohort study of all patients undergoing RHCM at Northumbria Healthcare NHS Foundation Trust. The first RHCM was performed in April 2022, and the final patient in this cohort underwent their operation in January 2023; during this period all patients undergoing elective surgery for achalasia underwent surgery with a robotic approach. Preoperative workup for all patients included upper gastrointestinal endoscopy, contrast swallow and oesophageal physiology studies to confirm a diagnosis of achalasia. The robotic platform used was the DaVinci Xi (Intuitive Surgical Inc., Sunnyvale, CA, USA). All operations were performed by two senior laparoscopic upper gastrointestinal surgeons, with over 50 years’ combined consultant experience, both of whom completed the required intuitive induction and proctoring pathways. Local hospital trust approval for the introduction of the robotic upper GI surgery programme was obtained prior to the first robotic operation. Additional specific institutional review board approval was not undertaken for this cohort of patients undergoing RHCM.

**Figure 1:**
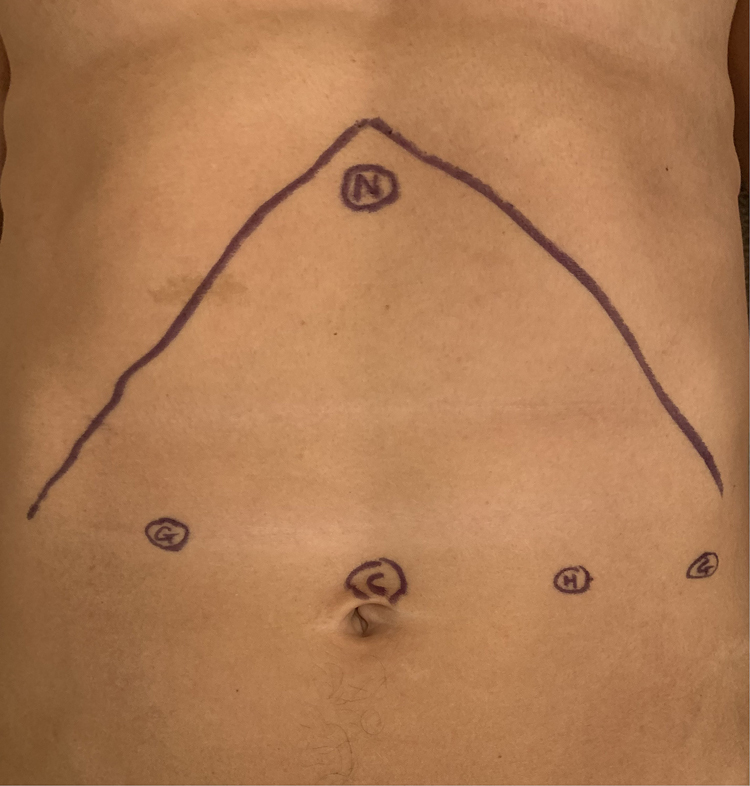
Port positions for robotic Heller’s cardiomyotomy. C = camera port; G = Cadière graspers; H = hook; N = Nathanson liver retractor

Patients are positioned in the reverse Trendelenburg position with their arms wrapped by their sides. Intraoperative endoscopy to clear oesophageal debris was not performed, as per the unit’s policy. A four 8mm port technique is used. The camera port is placed via an open trans-umbilical approach; two further ports are placed in the left upper quadrant, and the last is placed in the right upper quadrant. A 30-degree camera is used. A Nathanson liver retractor is used to allow access to the gastro-oesophageal junction. An AirSeal® Intelligent Flow System is used for insufflation. The intra-abdominal pressure is set at 6–8mmHg; the authors believe the use of low pressure is likely to reduce postoperative pain attributable to insufflation.^24^ Two Cadière forceps are used in the lateral ports, and a diathermy hook in the medial port is used to perform the myotomy. Port positions can be seen in Figure 1. The right crus is identified after division of the gastro-hepatic ligament. Anterior exposure of the oesophagus is achieved by dividing the overlying phreno-oesophageal ligament. Circumferential exposure of the oesophagus is not required. Mediastinal dissection of the anterior oesophagus is continued to expose an adequate oesophageal length for the myotomy. The anterior vagal nerve is identified and protected, so as to avoid inadvertent vagal injury.

**Figure 2:**
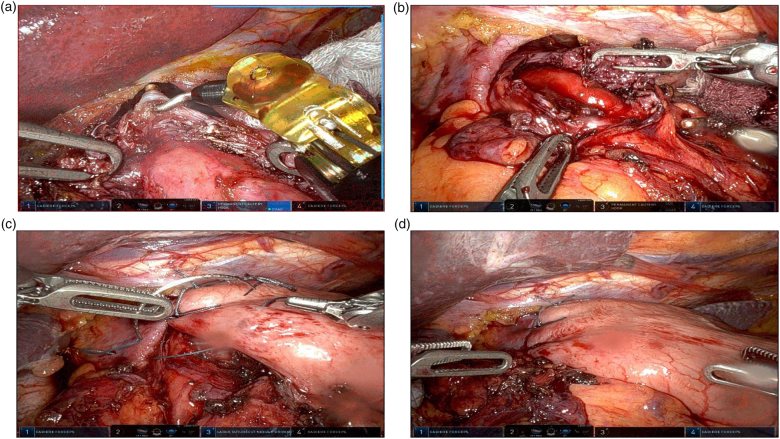
Intraoperative photographs of robotic Heller’s cardiomyotomy. (a) Hook diathermy to perform the myotomy. (b) Complete myotomy. (c) Performing anterior fundoplication. (d) Complete procedure.

The myotomy is performed between 11 and 1 o’clock on the oesophageal surface using the hook. The myotomy is extended at least 5cm above the gastro-oesophageal junction, and then a minimum of 2cm below (see Figure 2a and b). Care is taken to achieve division of all longitudinal and circular muscle fibres to ensure complete oesophageal mucosal billowing without perforating. The authors do not routinely perform endoscopy, or methylene blue testing, to check for perforation. The authors also perform an anterior 180-degree Dor fundoplication to reduce the risk of postoperative reflux.^25^ The short gastric arteries are divided only if necessary. Polydioxanone sutures are used to secure the wrap to the left crus, taking the muscle layers of the left side of the myotomy, and left crus together. Further sutures take the oesophageal muscle fibres of the right side of the myotomy, and the right crus (see Figure 2c and d). This covers the site of the myotomy, reducing the chance of a significant leak from a missed oesophageal enterotomy.

All relevant theatre timings were collected prospectively by the theatre staff. Total operating room time was defined as the time when the patient arrived in the anaesthetic room to the point of patient extubation. Docking time was defined as the time between the first skin incision to the time when the operating surgeon takes control of the robotic console. Console time is defined as the time between the first and last operative movement on the operative console.

Patients are all managed in a level 1 setting in the postoperative period. Routine postoperative contrast studies are not organised, nor is routine nasogastric drainage utilised. Patients are given routine postoperative antiemetics, and are advised to adhere to a liquid diet on the day of the operation and progress to soft or pureed diet on the first postoperative day. Where possible, it is policy in this unit to discharge patients on the same day of surgery.

Further advice and follow-up is provided by an upper GI specialist nurse who contacts the patient by phone at 48 hours following their operation, and sees them in a specialist clinic at two weeks. The consultant surgeon reviews the patient at six weeks postoperatively.

Resolution of the symptoms of achalasia were quantified using the Eckhard score, which was calculated after their symptoms were assessed in the postoperative clinic or telephone follow-up. A score of less than 3 indicates disease remission. The patient’s electronic regional general practitioner records and emergency department attendances were assessed on postoperative day 30 to assess early postoperative complications.

## Results

Thirteen patients underwent RHCM during this study period. During this time, no patients were excluded from the study. Ten patients were male and three were female. The median age was 56 years (range 20–80). Three patients were ASA 1, six patients were ASA 2 and four patients were ASA 3. For twelve patients, this was their first operative procedure for achalasia, and one patient underwent a revisional operation after previously having undergone a LHCM.

All operations were completed robotically with no patients requiring conversion to open. There were no intraoperative oesophageal perforations. Eleven patients had an anterior Dor fundoplication. Fundoplication was not possible in one patient due to left lobe hepatomegaly. The additional patient who was having revisional surgery did not undergo fundoplication in order to reduce the risk of iatrogenic injury due to hostile operative conditions and hiatal scarring.

**Figure 3:**
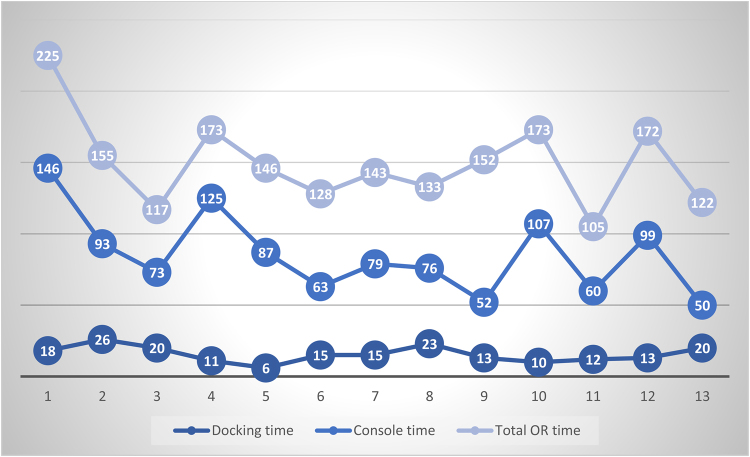
Operative timings (minutes) of the 13 consecutive patients undergoing robotic Heller’s cardiomyotomy. OR =operating room

Operative timings can be seen in Figure 3. Other than the first procedure, total operating room time was less than three hours for all patients. Docking time ranged from 6 to 26 minutes, and console time ranged from 50 to 146 minutes.

Six patients were discharged as day cases, six patients were discharged on the first postoperative day and one patient’s hospital stay was two nights. There was only one perioperative complication, which was urinary retention. In the first 30 days of follow-up, there was no perioperative mortality or re-operations. Two patients attended the surgical assessment unit during the follow-up period. One was diagnosed with oral thrush on postoperative day 6 and was discharged with topical therapy. The second presented with abdominal distention and pain on postoperative day 4. They underwent computed tomography investigation, which excluded any surgical complication, and they were discharged with laxatives.

All patients reported improvement of postoperative symptoms when compared with preoperative symptoms, and all had a postoperative Eckhard score of less than 3, indicating their achalasia was in remission. Five patients had an Eckhard score of 0, seven reported a score of 1, and one reported a score of 2. Six patients had ongoing mild dysphagia, one reported a small amount of weight loss while in the immediate postoperative period, one reported occasional retrosternal chest pain and one reported occasional regurgitation; all patients stated that all of their symptoms had improved following surgery.

## Discussion

This is the largest presented UK cohort of patients with achalasia who have undergone RHCM in a single NHS institution. The authors have demonstrated an excellent safety profile, with no cases of oesophageal perforation, a satisfactory learning curve and excellent patient outcomes.

Literature from the USA, where the adoption of robotics surgery has been higher, has also demonstrated that RHCM has an excellent safety profile. Multiple publications have demonstrated an absence of oesophageal mucosal perforation in their cohorts when using the robotic approach. Melvin and colleagues presented a prospective series from Ohio of 104 patients who underwent a RHCM without a single oesophageal perforation.^26^ Gharagozloo *et al* reported no mucosal perforations in their cohort of 48 patients from Florida.^27^ A further prospective series from Ohio reported 0/24 patients who had RHCM sustained oesophageal perforation, compared with 3/37 who underwent LHCM.^28^ An additional large retrospective study of patients from Ohio demonstrated an absence of mucosal perforation during 56 RHCMs, and compared that with 3/19 patients who had a LHCM.^23^ Furthermore, Horgan *et al* demonstrated no perforations in 59 patients who had undergone RHCM; the same authors reported a perforation rate of 10/62 following LHCM.^22^ A study from Texas demonstrated that 0/44 patients who underwent RHCM experienced complications, whereas 7/40 patients who underwent LHCM experienced complications.^21^

Although oesophageal perforation does not result in inferior symptom resolution following cardiomyotomy,^29^ the impact that perforation has on the individual patient in the short term is significant. As RHCM has consistently been demonstrated to have low rates of oesophageal perforation, the present authors believe that patients should be offered a robotic approach in favour of the traditional laparoscopic approach for cardiomyotomy.

RHCM has been reported to be at least non-inferior with regards to postoperative symptom resolution when compared with LHCM. Multiple publications have demonstrated that there is no difference in long-term postoperative rates of dysphagia or reintervention.^19,20,22,23^ However, Raja *et al* reported that patients undergoing RHCM had better postoperative function, with regards to complete oesophageal emptying, and lower reintervention rates when compared with LHCM.^30^ Kim *et al* also concluded that patients who had RHCM had less chance of symptom recurrence when compared with LHCM (0/37 vs 4/35); the authors once again attributed this benefit to the longer myotomy achievable using a robotic technique.^31^

The uptake of robotic surgery has been slower in European populations when compared with North American cohorts; this has largely been attributed to the increased cost of robotics.^32–34^ A retrospective review of the USA National Inpatient Sample of patients undergoing RHCM was associated with increased cost (US$42,900 vs US$34,300) when compared with LHCM.^35^ Another publication from the USA has also demonstrated that LHCM is significantly cheaper than RHCM (US$7,441±7,897 vs US$9,415±5,515).^34^ Although cost reduction strategies are likely to arise with the increasing utilisation of robotics,^32,34,36^ at present the robotic approach should be considered to be more expensive.

Another barrier for adoption of robotic surgery is the perceived increase in operative timings. The present study has demonstrated satisfactory operative times in this early cohort. These timings are comparable to that of the published literature where it has been reported that RHCM does not take significantly longer than LHCM,^19,23,37^ and operative times improve with surgeon experience.^22,37^

Other published literature does not report significant differences in length of stay (LOS) when comparing RHCM with LHCM.^19–23,34,35^ However, in the present cohort, 12/13 patients were discharged as either day cases or had a single overnight stay. We believe that the reduced risk of oesophageal perforation has increased our team’s confidence in early discharge of these patients. This day case approach will also inevitably reduce the cost associated with RHCM.

Despite these perceived barriers to the robotic approach for the management of achalasia, there have been a small number of published European cohorts. A high-volume Swiss centre reported outcomes following 11 RHCM. They demonstrated comparable results with regards to patient satisfaction, LOS and complication rates when compared with their LHCM results. Importantly, they too had no oesophageal perforations in their robotic cohort.^18^ A small cohort of five patients who underwent RHCM at a London hospital also demonstrated symptom improvement in all five patients, including one patient who experienced oeosphageal mucosal injury.^38^ A further Turkish cohort of six patients reported excellent postoperative patient satisfaction following RHCM, and the absence of oesophageal perforation.^39^

The largest published European cohort of patients undergoing RHCM comes from a regional oesophageal referral centre in Italy. They report the outcomes of their first 69 patients who underwent RHCM for achalasia across an 8-year period. Once again, they demonstrated no mucosal perforations in all patients.^40^

The evidence from the present study, and from the published literature confirms that the robotic approach should continue to be adopted by European surgeons. It results in fewer episodes of oesophageal perforation. At present there is only a single perforation recorded in the published cohorts across Europe and North America.^38^ The present data, from the second largest reported European cohort of patients undergoing surgery for achalasia, has demonstrated that transition to RHCM is feasible, and produces excellent early results. The authors believe that the robotic approach significantly improves the safety profile of this procedure. It is likely that the improved 3-dimensional optics, instrument stability and degree of movement achieved by the robotic system is the principal reason for this.

Although this is the second largest reported European cohort of patients who have undergone RHCM, it includes only 13 patients; therefore, there is a chance that the absence of oesophageal injury in our cohort is due to low sample size; however, the published literature consistently demonstrates the absence of perforations in RHCM.

## Conclusion

This is the second largest reported European cohort of patients who have undergone robotic Heller’s cardiomyotomy. The Northumbria Upper Gastrointestinal Robotic team has demonstrated that RHCM has an exceptional safety profile. Total operative time is comparable to the published laparoscopic approach. The robotic approach may be superior to laparoscopy as it allows more precise identification and dissection of the oesophageal muscles, which likely reduces the risk of inadvertent mucosal damage. Although at present the robotic approach is likely to be more expensive than the laparoscopic approach, RHCM has an improved safety profile which has been repeatedly highlighted in the literature and is reflected in our first 13 patients.
